# DNA-functionalized gold nanoparticles: Modification, characterization, and biomedical applications

**DOI:** 10.3389/fchem.2022.1095488

**Published:** 2022-12-13

**Authors:** Xiaoyi Ma, Xiaoqiang Li, Gangyin Luo, Jin Jiao

**Affiliations:** ^1^ Suzhou Institute of Biomedical Engineering and Technology, Chinese Academy of Sciences, Suzhou, China; ^2^ School of Life Sciences, Shandong First Medical University and Shandong Academy of Medical Sciences, Jinan, Shandong, China

**Keywords:** DNA modification, biosensor, DNA hybridization, gold nanoparticles, therapy

## Abstract

With the development of technologies based on gold nanoparticles (AuNPs), bare AuNPs cannot meet the increasing requirements of biomedical applications. Modifications with different functional ligands are usually needed. DNA is not only the main genetic material, but also a good biological material, which has excellent biocompatibility, facile design, and accurate identification. DNA is a perfect ligand candidate for AuNPs, which can make up for the shortcoming of bare AuNPs. DNA-modified AuNPs (DNA-AuNPs) have exciting features and bright prospects in many fields, which have been intensively investigated in the past decade. In this review, we summarize the various approaches for the immobilization of DNA strands on the surface of AuNPs. Representative studies for biomedical applications based on DNA-AuNPs are also discussed. Finally, we present the challenges and future directions.

## 1 Introduction

Gold nanoparticles (AuNPs) are tiny particles of gold with a size of 1–100 nm (Cao et al., 2022). AuNPs have attracted extensive attention due to their special physical and chemical properties (Zha et al., 2021), which are different from the counterpart bulk materials and small molecules. As early as the fourth century, AuNPs were introduced into the production of glass to make the well-known Craggs cup, which can display red or green depending on how it is exposed to the light. In 1857, Faraday discovered that some fine particles could be obtained by treating aqueous solutions of chloroauric acid with a two-phase system dissolved in phosphorus and carbon disulfide (Faraday, 1997). The water suspension of these fine particles displays beautiful wine red, which completely differs from the block gold. Because of technical restrictions and the incompleteness of the AuNPs studies at the time, this is the first published article regarding the preparation of AuNPs.

With the development of nanotechnology, novel physicochemical properties of AuNPs are being discovered, e.g., high extinction coefficients in the visible region (Deng et al., 2014), fluorescence quenching effect (Zhang et al., 2017), huge specific surface area (Wall et al., 2017), facile synthesis (Zhang et al., 2022a), and strong localized surface plasmon resonance (LSPR) absorption (Ghosh and Pal, 2007). These properties make AuNPs have wide applications in the biomedical field (Miao and Tang, 2020a; Chai et al., 2021). For example, because the LSPR of AuNPs is highly sensitive to the size (Lee and El-Sayed, 2006; Tan et al., 2017), capping molecule (Chen et al., 2017), morphology (Burda et al., 2005; Liu et al., 2014), medium refractive index (Underwood and Mulvaney, 1994), and distribution state (Ma et al., 2018; Ding et al., 2019), they have been applied to different colorimetric biosensors (Ma et al., 2017; Ma et al., 2018). Additionally, because of the excellent fluorescence quenching ability, AuNPs have been used to develop various fluorescent biosensors for biomolecular analysis (Liu et al., 2018a; Miao et al., 2018). Moreover, AuNPs have several advantages over other nanomaterials that make them particularly status in the biomedical field (Zhang et al., 2019a; Ma et al., 2020). First, the synthesis of AuNPs is extremely simple and does not require expensive equipment (Frens, 1973; Brust et al., 1994). Second, raw materials for synthesizing AuNPs are easily available and cheap, making the application of AuNPs in the biomedical field extremely cost-effective (Wang et al., 2015). Third, the size, shape, aggregation state, and surface modification of AuNPs can be finely tuned, which is crucial in constructing biosensors (Brust et al., 1994). Finally, the biocompatibility of AuNPs is usually better than that of many other nanomaterials. These characteristics allow researchers to develop novel tools for the biomedical field (Miao et al., 2017; Zhang et al., 2022a). Nevertheless, naked AuNPs cannot meet the requirements of multifunctional biological applications. Certain limitations exist, such as poor stability in high-salt solutions, weak specificity, and sensitivity.

Deoxyribonucleic acid (DNA) is an organic compound with complicated molecular structures; it exists in the eukaryotic nucleus as a vital component of chromosomes. Since the 1980’s, DNA has become known as the vehicle for the organism’s genetic information. Afterward, Seeman and Sleiman. (2017) proposed that DNA is not only a genetic material but also performs some special functions due to its precise base pairing. Since then, DNA self-assembly technology based on base complementary pairing has developed rapidly (Jiang et al., 2021a; Miao and Tang, 2021). With the development of DNA technology, novel DNA functions have been found (Heuer-Jungemann and Liedl, 2019; Miao and Tang, 2020b; Miao and Tang, 2020c). Owing to the outstanding properties of DNA (Miao and Tang, 2021), such as excellent biocompatibility (Liu et al., 2022a), facile design (Chai et al., 2022a), and accurate identification (Duskova et al., 2020; Li et al., 2021), it has been applied in various fields (Aizitiaili et al., 2021; Jiang et al., 2021b; Mao et al., 2022).

DNA can perfectly make up for the shortcoming of bare AuNPs. Researchers have anchored various DNA probes on the surface of AuNPs, and studies have shown that the surface of AuNPs can form strong covalent bonds with thiol groups, readily. Afterward, the surface modification of AuNPs is usually based on the formation of Au–S bonds. DNA-modified AuNPs (DNA-AuNPs) are highly stable and have diverse functionalization options (Zhou et al., 2022a; Chen et al., 2022). Moreover, modified nanomaterials exhibit properties that differ from those of both the AuNPs and DNA from which they derive. DNA-AuNPs have two highlights: 1) they provide novel physical and chemical properties to each other (e.g., plasmonic (Liu et al., 2022b), catalytic (Yang et al., 2018), scattering (Wang et al., 2022), and quenching (Li et al., 2020)), which are paramount in the contexts of material design and probe design; 2) the nanomaterials can be employed as a scaffold for probe-assembling and concentration into a dense arrangement that gives rise to many of their functional properties and reaction efficiency due to the DNA modification of AuNPs (Xie et al., 2019), which offers more possibilities for their application in the biomedical field (Tang et al., 2018).

## 2 DNA modification of AuNPs

The DNA-AuNPs’ application in the biomedical field has emerged since 1996 with the landmark work for attaching thiol DNA to AuNPs, which was proposed by Mirkin et al. (1996) This proposed strategy made numerous applications of DNA-AuNPs possible, including the preparation of biosensing (Pan et al., 2019), bioimaging (Sun et al., 2019a; Zhou et al., 2022b), disease diagnosis (Lin et al., 2019), and drug delivery (Zheng et al., 2019). In brief, the simple and efficient modification methods of DNA-based AuNPs play a crucial role in all these applications (Degliangeli et al., 2014; Peng et al., 2022). With the exploration of the physicochemical properties of AuNPs, several modification methods that provide more convenient conditions for the DNA-AuNPs have been reported. To date, although several reviews have been presented to introduce the synthesis methods and application of AuNPs in biosensing and bioimaging, none of them has focused on the modification methods of AuNPs. Hence, we present a review of the modification methods of AuNPs in recent years to summarize and comment on their development and advance.

### 2.1 The salt-aging method

**FIGURE 1:**
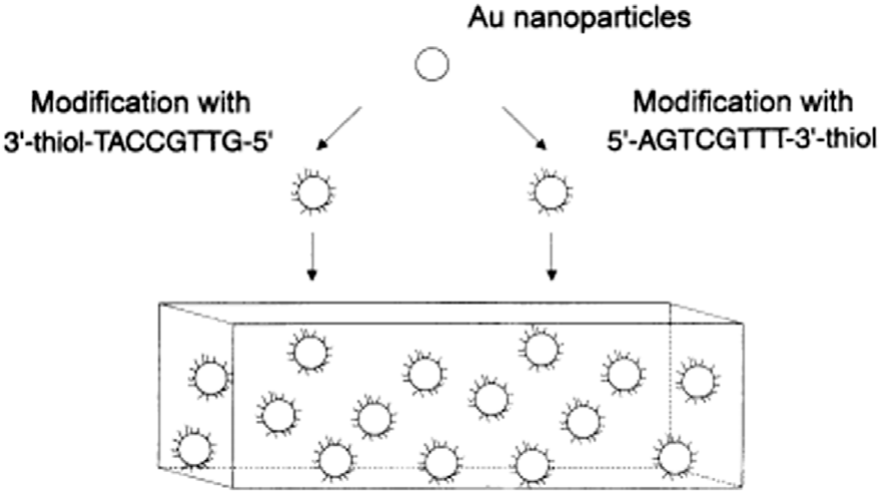
Illustration of the salt-aging method for DNA-AuNPs. Reprinted with permission from ref 50.

DNAs are strongly negatively charged and cannot pack densely without electrostatic screening. Mirkin et al. (1996) developed a method to prepare DNA-AuNPs that introduced the concept of salt-aging, which allows for high-density packing of thiol DNA on the AuNPs’ surface. This method remains the preferred one for the preparation of DNA-AuNPs. As shown in Figure 1, an excess amount of DNA and a high concentration of NaCl must be present, and NaCl is gradually added to increase DNA loading over 1–2 days. According to the literature, increasing the sodium ion concentration of the reaction solution to >0.15 M (up to ∼2.0 M with surfactants) screens the repulsive interactions between neighboring strands, thereby promoting higher densities as DNA assembles on the surface of AuNPs. Higher salt concentrations generally result in higher DNA densities until steric constraints prohibit further adsorption. The high loading of DNA on the AuNPs’ surface derives from 1) the substitutionally labile coordination sphere of citrate ions adsorbed onto the surfaces of the particle precursors used to prepare the DNA-AuNPs, 2) the thiol–Au chemistry used to surface immobilize the DNA, and 3) the salt-aging procedures used to decrease electrostatic interactions between neighboring DNA. In summary, the monolayer of DNA formed by this method is particularly stable because of the relatively strong Au–S interaction (compared with the Au–citrate interaction).

### 2.2 Bromide as robust backfiller on the AuNPs

**FIGURE 2:**
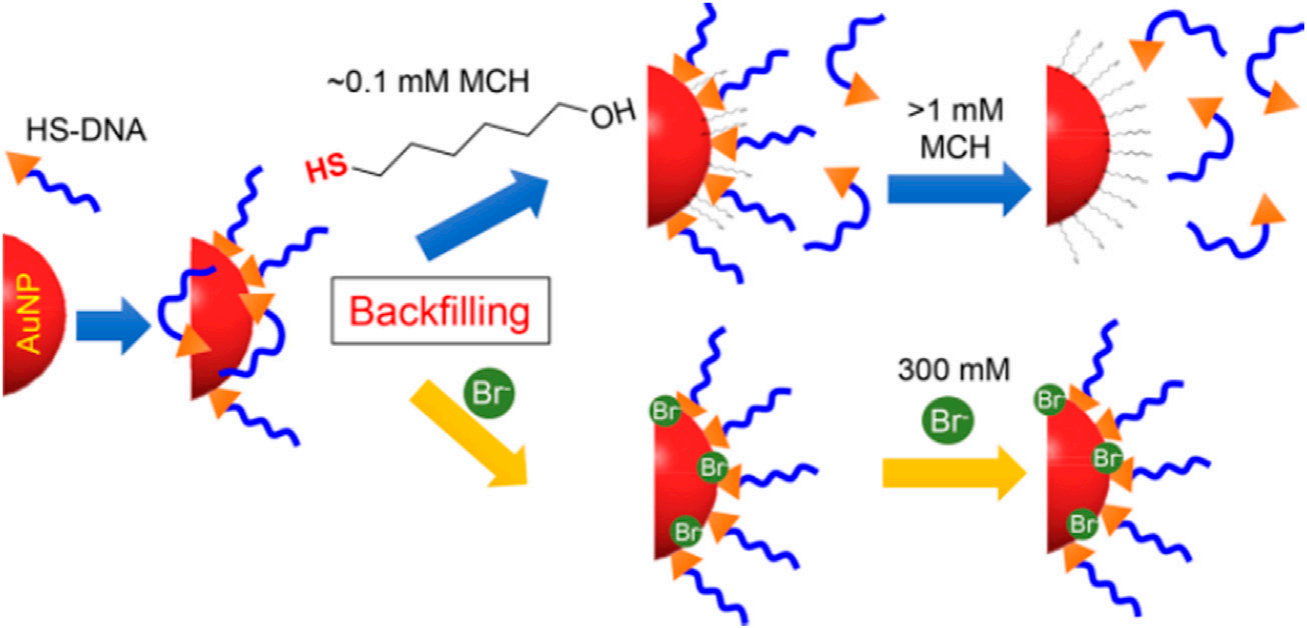
Illustration of bromide as a robust backfiller on the AuNPs for synthesizing DNA-AuNPs. Reprinted with permission from ref 58.

Unintended DNA absorption is difficult to be avoided, which reduces the DNA hybridization effect and weakens the colloidal stability of the conjugate. Hence, another backfiller that absorbs weaker than the thiol but stronger than certain bases may allow precise control of DNA conformation for a better modification effect. According to Martin et al. (Mirkin et al., 1996), halides containing F^−^, Cl^−^, Br^−^, and I^−^ have been tested for exploring more controllable backfillers (Figure 2). By examining various sizes of AuNPs, it was found that Br^−^ improved not only colloidal stability but also hybridization efficiency (Liu et al., 2018b). Additionally, NaBr was used as a backfiller to experiment on AuNPs with sizes from 5 to 80 nm and different DNA sequences to realize excellent results. In this regard, Br^−^ can be considered as a backfiller that can more precisely control the DNA conformation on AuNPs. With this strategy, a novel and excellent assisted material for salt-aging method has emerged.

### 2.3 The vacuum centrifugation method

AuNPs conjugated with thiol DNA represent an attractive and alternative platform for applications in biosensors, medical diagnostics, and biological analyses. However, the DNA-AuNPs used in the above methods are time-consuming and need a 2 days incubation process to achieve a high DNA loading density. Considering the time-consuming feature of classic approaches, alternative methods that can shorten the time required while the quality of the DNA-AuNPs is preserved or improved are needed. An approach reported by Brust et al. (Kanaras et al., 2003) needs 20 h for the entire modified process. This method employs vacuum centrifugation instead of the aging step, which shortens the labeling time. In this method, the thiol DNA is incubated with AuNPs overnight, and the solution is diluted to a final volume of 1 ml containing NaCl (0.1 M) and sodium phosphate buffer (5 mM, pH 7.0) solutions. After further incubation for 2 h, the volume was slowly reduced to 150 µl by vacuum centrifugation over 3–4 h at 40°C. This is an important process that can ensure a gradual and simultaneous increase in ionic strength and DNA concentration, which can extremely stabilize DNA-AuNPs. This approach has great significance in developing the fast and reproducible synthesis of stable DNA-AuNPs.

### 2.4 BSPP coating approach

A novel strategy was developed by Alivisatos et al. (Claridge et al., 2005), which utilized a polymeric layer (bis (*p*-sulfonatophenyl)phenylphosphine dehydrate dipotassium salt, BSPP) to stabilize AuNPs before adding thiol DNA. The modification time is reduced to 12 h. In this strategy, BSPP is mixed with AuNPs to allow phosphine ligands to replace the citrate ligands. AuNPs with a diameter of 5 or 10 nm, which can form a complex with BSPP did not aggregate in aqueous buffers and could be subjected to repeated cycles of NaCl precipitation. Meanwhile, the thiol groups were incorporated into DNA, which can penetrate the ligand “shells” and react directly with the surface of AuNPs. Under the BSPP protection, the reaction efficiency was improved and the labeling time was decreased.

### 2.5 The modification method assisted by dATP

**FIGURE 3:**
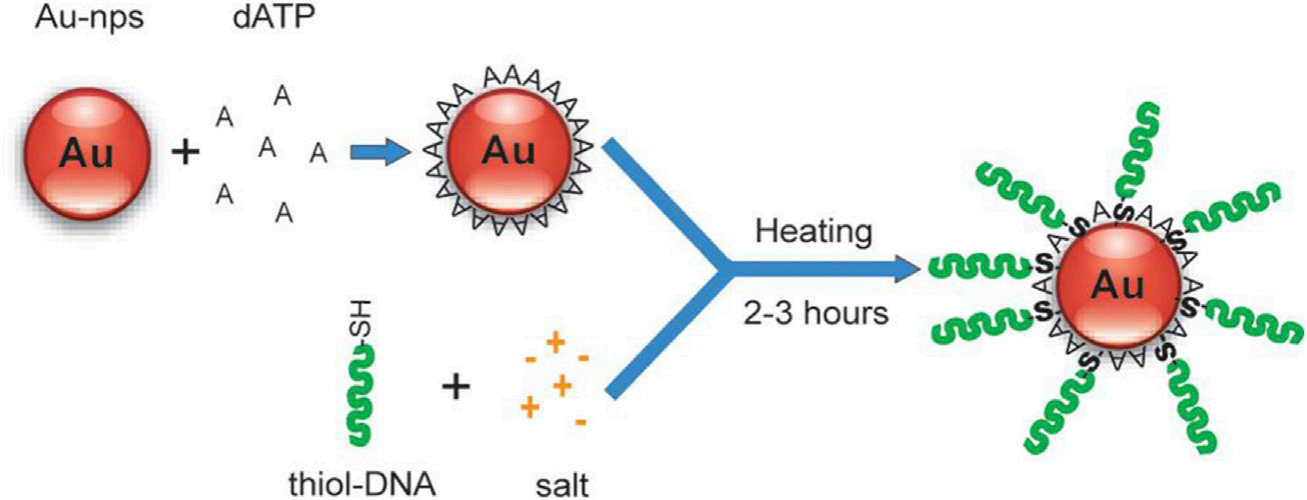
Mononucleotide-assisted conjugation of thiol DNA to AuNPs. Reprinted with permission from ref 61.

With BSPP protection, the salt-dependent aggregation of AuNPs was controlled, which decreased the labeling time but was not intended for preparing DNA-AuNPs with a high DNA loading density. Another approach for DNA-AuNPs construction was then proposed by Hsing et al. (Zhao et al., 2009) This strategy enabled rapid immobilization of high-density DNA on stable AuNPs in a salt environment. The strategy relied on fast and reversible binding of mononucleotide to AuNPs’ surfaces, which was used to quickly stabilize AuNPs in a salt solution by forming a mononucleotide layer on the AuNPs’ surface. The adsorption/desorption of this layer is thermally tunable. Figure 3 shows that the modified process starts with the reversible adsorption of dATP on the AuNPs for nanoparticle stabilization in salt solutions, followed by a temperature-facilitated ligand exchange reaction between the incoming thiol DNA and the protection dATP on the AuNPs’ surface. The synthesis of DNA-AuNPs using this approach could be achieved within several hours and DNA-AuNPs have similar or better characteristic attributes compared with those synthesized using conventional methods. The ability to modify AuNPs with specific DNAs is an efficient and improved application in molecular diagnostics and biomedical fields.

### 2.6 The synthesis method based on tween 80

**FIGURE 4:**
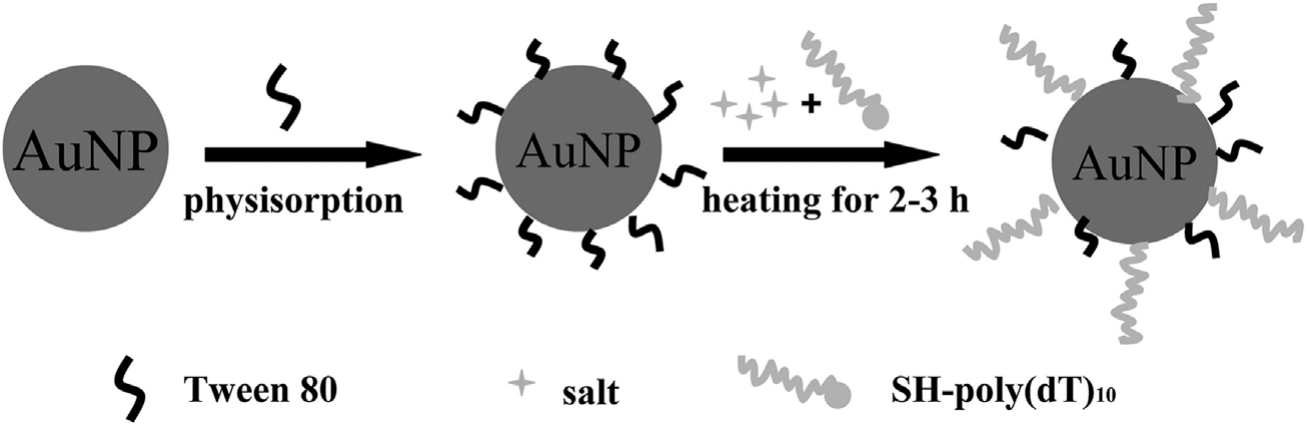
Schematic representation of the principle of Tween 80-mediated conjugation of thiol DNA to AuNP. Reprinted with permission from ref 62.

Another simple and convenient method was presented by Wu et al. (Xu et al., 2011). This method exploited the stabilizing effect of Tween 80 on AuNPs in a salt solution and a heating-facilitated ligand exchange between Tween 80 and thiol DNA. Before the addition of thiol DNA, Tween 80 was added and adsorbed onto the AuNPs’ surfaces, which could effectively stabilize AuNPs in salt solutions by forming a protection layer on the AuNPs’ surfaces. Under the protection of Tween 80, DNA-AuNPs could be rapidly formed in a salt solution followed by a mild heating-facilitated ligand exchanged between Tween 80 and thiol DNA (Figure 4). Meanwhile, with this method, DNA-AuNPs can be completed within 2–3 h, and has similar or better quality in terms of DNA loading density and stability than the classic methods.

### 2.7 The freezing method

**FIGURE 5:**
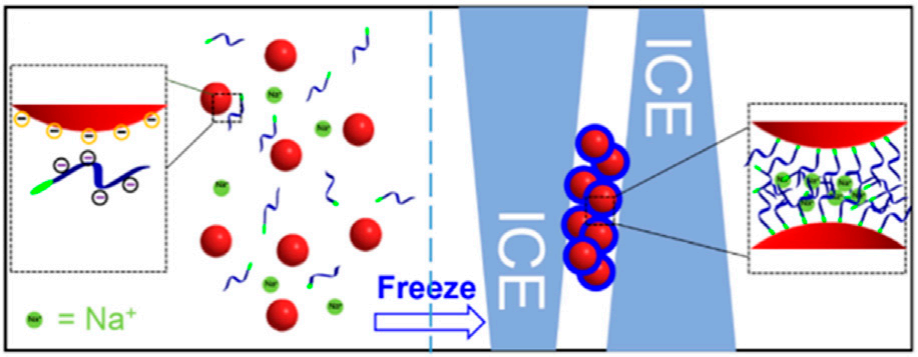
The principle of the freezing method that can attach thiol DNA onto the surface of AuNPs. Reprinted with permission from ref 63.

Nanoparticle solutions cannot be frozen, which causes irreversible aggregation. This is because salt and nanoparticles are excluded by the growing ice crystals that cause the local salt concentration to increase and screen the electrostatic repulsion between the nanoparticles (Deville, 2017). However, the nanoparticles are modified by some macromolecules, and the irreversible aggregation might be reversible because of the steric stabilization between the nanoparticles and macromolecules. According to this theory, a novel and easy-to-operate method was proposed by Liu et al. (Liu and Liu, 2017) (Figure 5). According to the report, after mixing the appropriate concentration of DNA with AuNPs, the mixture was placed in a laboratory freezer for 2 h or less time, followed by thawing at room temperature. The thiol DNA can be modified on the surface of AuNPs with high DNA density. This proposed method also applies to various sizes of AuNPs. Meanwhile, this method has the following merits. First, extra reagents are not required. Second, higher DNA density with higher colloidal stability compared with other methods. Although the other methods have driven the development of this field in so many years, this method brought novel features that will enable more applications in the biomedical field.

### 2.8 The two-step method based on mercaptoethanol

**FIGURE 6:**
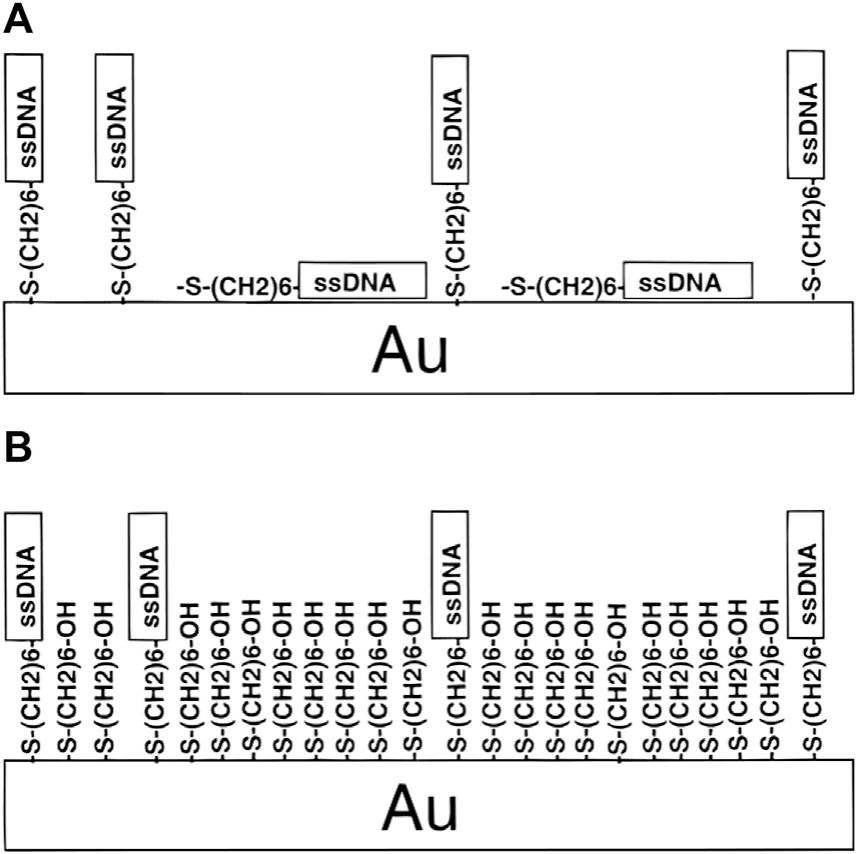
Schematic of the two-step method based on mercaptoethanol. Reprinted with permission from ref 64. **(A)** HS-ssDNA on AuNPs and **(B)** both HS-ssDNA and MCH adsorbed on AuNPs.

Afterward, a novel approach for thiol DNA-AuNPs was proposed by Herne et al. (Herne and Tarlov, 1997). They used alkanethiol self-assembly methods to fabricate DNA-AuNPs with known and reproducible probe coverages, and showed high hybridization activity. Figure 6 shows that the surface coverage of thiol-derivatized DNA on the surface of AuNPs can be precisely controlled by forming mixed monolayers of the thiol-derivatized DNA and MCH and containing the spacer thiol that was carefully chosen to minimize non-specific absorption of single-stranded DNAs. This strategy not only provides a novel method for synthesizing DNA-AuNPs but also shortens the labeling time; only 1 h is required for the entire modified process.

### 2.9 pH method

**FIGURE 7:**
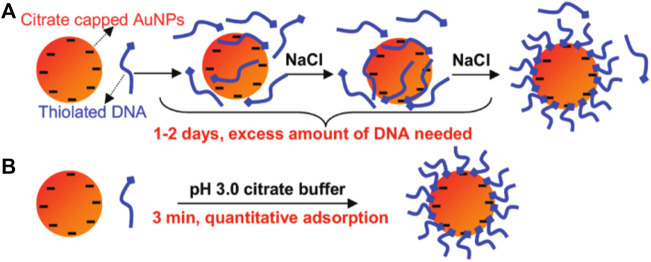
Scheme showing the adjusted pH method. Reprinted with permission from ref 65. **(A)** Traditional method and **(B)** low PH-assisted method.

In the above methods, the assisted molecules must be gradually added to the complex of DNA and AuNPs over 1–2 h every 1–2 days, which is somewhat time-consuming. Hence, an approach for fast, convenient, and universal DNA loading is required. Liu et al. (Zhang et al., 2012) developed a novel method using a pH 3.0 citrate buffer to complete the attachment process in a few minutes (Figure 7). The only required reagent is a low-pH citrate buffer. This approach is universal for various sizes of AuNPs. Another important feature of this approach is to achieve quantitative DNA density and the attachment of multiple DNAs at designated ratios. This work indicated that pH provides a novel and fast method to tune the interaction between DNA and AuNPs with an excellent effect.

### 2.10 Polyadenosinic acid-assisted method

**FIGURE 8:**
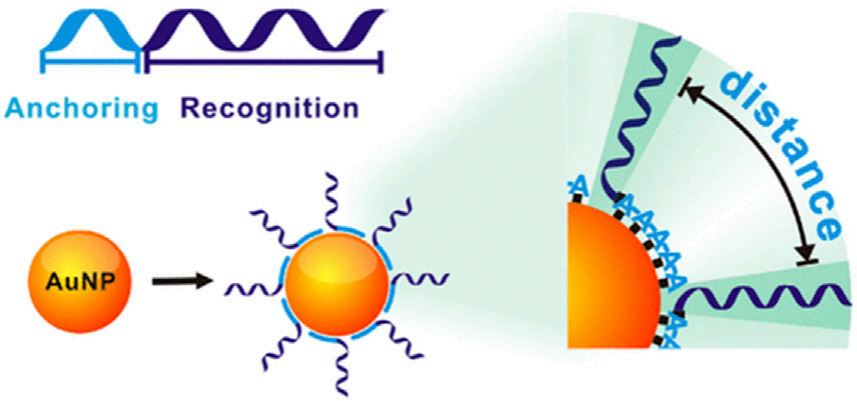
The principle of the ploy A-assisted method that can control on AuNPs by varying the length of poly A blocks. Reprinted with permission from ref 66.

The above methods are mainly based on strong Au–S chemistry to self-assemble thiol DNA at AuNPs However, precisely controlling the orientation and conformation of surface-tethered DNA and finely tuning its hybridization ability remain challenging. Fan et al. (Pei et al., 2012) developed a novel strategy for DNA-AuNPs free of DNA modifications (Figure 8). They demonstrated that poly adenine (ploy A) can serve as an effective anchoring block for preferential binding with the AuNPs’ surface, and the appended recognition block adopts an upright conformation that favors DNA hybridization. The lateral spacing and surface density of DNA on AuNPs can also be systematically modulated by adjusting the length of the poly A block. Significantly, this method results in DNA-AuNPs with high and tunable hybridization ability, which forms the basis of rapid plasmonic DNA biosensors.

## 3 Biomedical applications of DNA-AuNPs

AuNPs are widely used in the biomedical field due to their numerous outstanding properties. Furthermore, because of its programmable and sequence-specific interactions, DNA has emerged as a versatile and precise tool for biosensor (Chai and Miao, 2019), bioimaging (Meng et al., 2019), and drug delivery (Jiang et al., 2021b). DNA-AuNPs can overcome the shortcomings of naked AuNPs and endow AuNPs with promising applications in biomedical, such as the DNA-AuNPs being highly negatively charged, which improves the stability of AuNPs in complex samples. In summary, the DNA-AuNPs provide novel applications.

### 3.1 Biosensor

Biosensors are crucial analytical tools in the biomedical field, which uses the unique biophysical properties of materials to detect biomolecules. Biosensors include colorimetric biosensors (Hao et al., 2018; Chou et al., 2019), fluorescent biosensors (Liang et al., 2017; Jiang and Miao, 2020), and electrochemical biosensors (Jiang et al., 2021a; Chai et al., 2022b), which are differentiated by the output signal. DNA-AuNPs possess similar chemical and physical properties as naked AuNPs, which are endowed with better stability (Zhong et al., 2022) and functional diversity (Zhang et al., 2022b). Recently, many successful strategies for biosensors based on DNA-AuNPs have been proposed (Wang et al., 2020), and unexpected results have been achieved (Zhang et al., 2020).

#### 3.1.1 Colorimetric biosensor based on DNA-AuNPs

Colorimetric biosensors (Zhou et al., 2022c) measure the concentration of molecules by comparing the color changes in the solution of colored substrates. The Lambert–Beer law (Rose, 1952) explains that the extinction coefficient of substrates is directly related to the detection sensitivity. Hence, AuNPs are perfect candidates for colorimetric biosensors because they have an extremely high extinction coefficient (e.g., 2.7 × 10^8^ M^−1^ for 13 nm AuNPs), more than 1,000 times higher than those of organic dyes (Liu et al., 2007). As the modification methods of AuNPs mature, the explosive development of colorimetric biosensors has been reported for various species, including metal ions (Xie, 2018), nucleic acids (Karami et al., 2021; Zhu et al., 2021), proteins (Chen et al., 2016), and even cells (Zhang et al., 2014).

**FIGURE 9:**
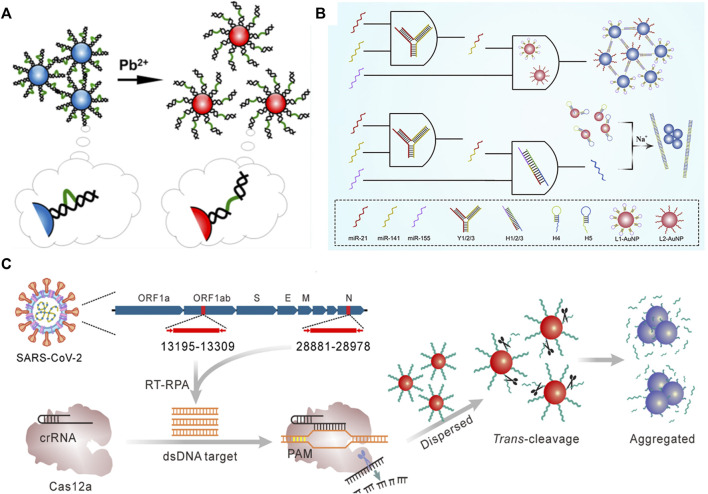
Illustration of the colorimetric biosensor detecting **(A)** Pb^2+^ **(B)** multiple miRNAs, and **(C)** gene. Reprinted with permission from refs 86–88.

For metal ion detection, Diao et al. (2020) developed a sensitive biosensor based on DNA-AuNPs for Pb^2+^ detection (Figure 9A). They used the LSPR of DNA-AuNPs to determine Pb^2+^ concentration. This colorimetric strategy with high selectivity had a detection limit of 8.0 nM, demonstrating excellent sensing performance. Furthermore, Ma et al. (2019) employed DNA-AuNPs as the signal output for multiple miRNA diagnostics (Figure 9B). They developed a novel logic gate based on DNA-AuNPs for triple miRNAs analysis. The key to achieving a DNA-AuNPs-based colorimetric biosensor is controlling the nanoparticle dispersion and aggregation, which can be realized by crosslinking using a linker DNA. To achieve accurate target capture, the triple miRNA inputs can initiate toehold-mediated strand displacement and generate linker DNA of two DNA-AuNPs, and the researchers specially designed a Y-shaped probe by hybridization of three DNA strands. Afterward, the LSPR absorption of AuNPs is used as the output. This method was successfully demonstrated in serum samples, which have remarkable potential applications in early cancer diagnosis and therapy.

Biosensors based on DNA-AuNPs can not only detect multiple nucleic acids but also achieve the detection of genes by the naked eye. As shown in Figure 9C, Zhang et al. (2021) reported a facile and cost-effective biosensor for severe acute respiratory syndrome coronavirus-2 (SARS-CoV-2). In this regard, reverse transcription recombinase polymerase amplification coupled with CRISPR-Cas12a colorimetric assay was proposed for SARS-CoV-2 detection. The researchers used DNA-AuNPs as a universal colorimetric readout, which can specifically target the ORF1ab and N regions of the SARS-CoV-2 genome. Notably, this strategy coupled with the isothermal amplification and Cas12a activation process can significantly improve the specificity.

#### 3.1.2 Fluorescence biosensor based on DNA-AuNPs

Fluorescent is known as an extremely sensitive technique that can even achieve single molecule detection sensitivity and plays a crucial role in biomedical diagnostics and biotechnology. However, many fluorescent assays face several challenges including the photostability and autofluorescence of biological samples. Recently, nanoparticle-based fluorescent biosensors have served as alternatives to traditional fluorescence dye-based assays. AuNPs have been demonstrated as highly efficient energy acceptors because of the continuous electron–hole pair excitation in the surface of AuNPs. Because of this property of AuNPs, novel fluorescent biosensors based on DNA-AuNPs have been developed for various molecule detection.

**FIGURE 10:**
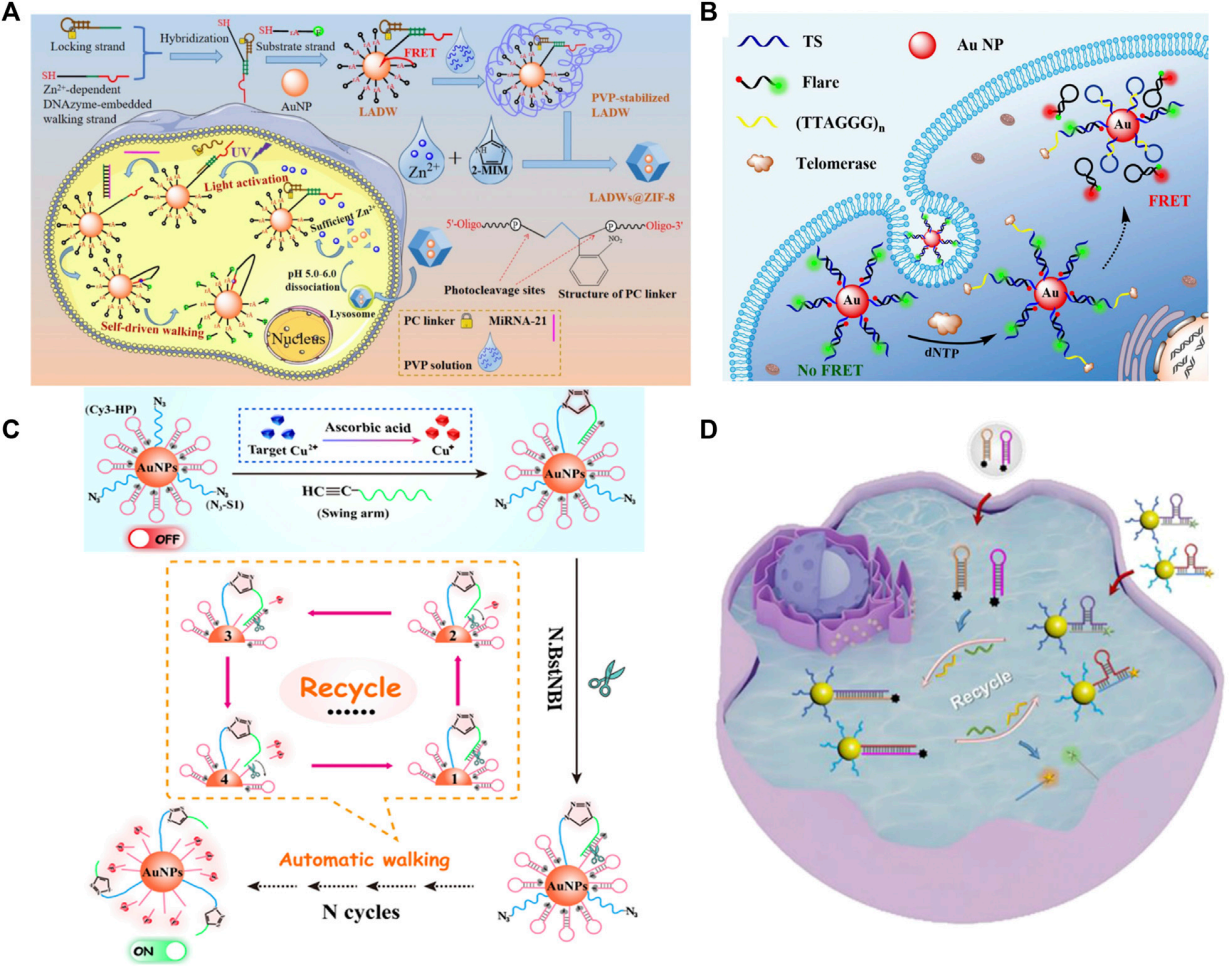
Illustration of a fluorescent biosensor based on DNA modified for detecting **(A)** miRNA, **(B)** telomerase activity, Cu^2+^, **(C)** multiple targets, and **(D)** multiple miRNAs *in situ* imaging. Reprinted with permission from refs 89–92.

Due to the design flexibility of DNA and excellent signal amplification ability, DNA-AuNPs have been widely applied to intracellular fluorescence imaging. Gao et al. (2021) demonstrated a novel fluorescent biosensor for miRNA-21 detection (Figure 10A). In this strategy, DNA-AuNPs were encapsulated within a dissociable zeolitic imidazolate framework-8 (ZIF-8) to facilitate endocytosis and ensure sufficient internal cofactors (Zn^2+^) to realize a self-driven pattern in the acidic environment of the cell lysosome. This strategy based on DNA-AuNPs presents satisfactory sensitivity and specificity. Importantly, the sensing platform could distinguish the variations of targets in living cancer cells with extremely high efficiency and exceptional precision, also offering a powerful tool for intracellular imaging.

Moreover, DNA-AuNPs can be used for enzyme activity detection. Yang et al. (2017) designed a fluorescent biosensor for intracellular telomerase detection. This strategy demonstrated a probe termed FRET nanoflare based on DNA-AuNPs to detect the activity of telomerase in living cells with extremely high specificity (Figure 10B). This approach can detect telomerase in cell lysate with the limit of detection (LOD) down to ∼33 Hela cells. Furthermore, *in situ* imaging and monitoring of intracellular telomerase is satisfactory.

Biosensors based on DNA-AuNPs are also used to detect intracellular metal ions by a series of chemical reactions. A highly selective and sensitive “OFF–ON” fluorescent biosensor designed for intracellular Cu^2+^ detection has been reported (Wang et al., 2021) (Figure 10C). This strategy depends on integrating the high selectivity of the Cu(I)-catalyzed click reaction with the ultrahigh sensitivity of a spherical nucleic acid-based three-dimensional (3D) DNA walker. Accordingly, the ingenious integration of an efficient click reaction and smart 3D DNA walker endows the constructed fluorescent biosensor with superior selectivity and ultrahigh sensitivity.

Biosensors based on DNA-AuNPs have a brilliant performance in single-target detection; they can also be applied to detect multiple targets. Xu et al. (2022) employed DNA-AuNPs as fluorophore/mass dual-encoded nanoprobes, which can execute target-triggered hairpin self-assembly to enable *in situ* amplified imaging and follow-up mass spectrometry (MS) quantification of intracellular multiple miRNAs (Figure 10D). Fluorescence imaging and MS quantification of miRNA-21 and miRNA-141 have also been accomplished in different cell lines, highlighting its potential in cell subtyping. This strategy creates a new approach for multiple intracellular miRNA detection and determining multiple biomarkers in the biomedical field.

#### 3.1.3 Electrochemical biosensor based on DNA-AuNPs

**FIGURE 11:**
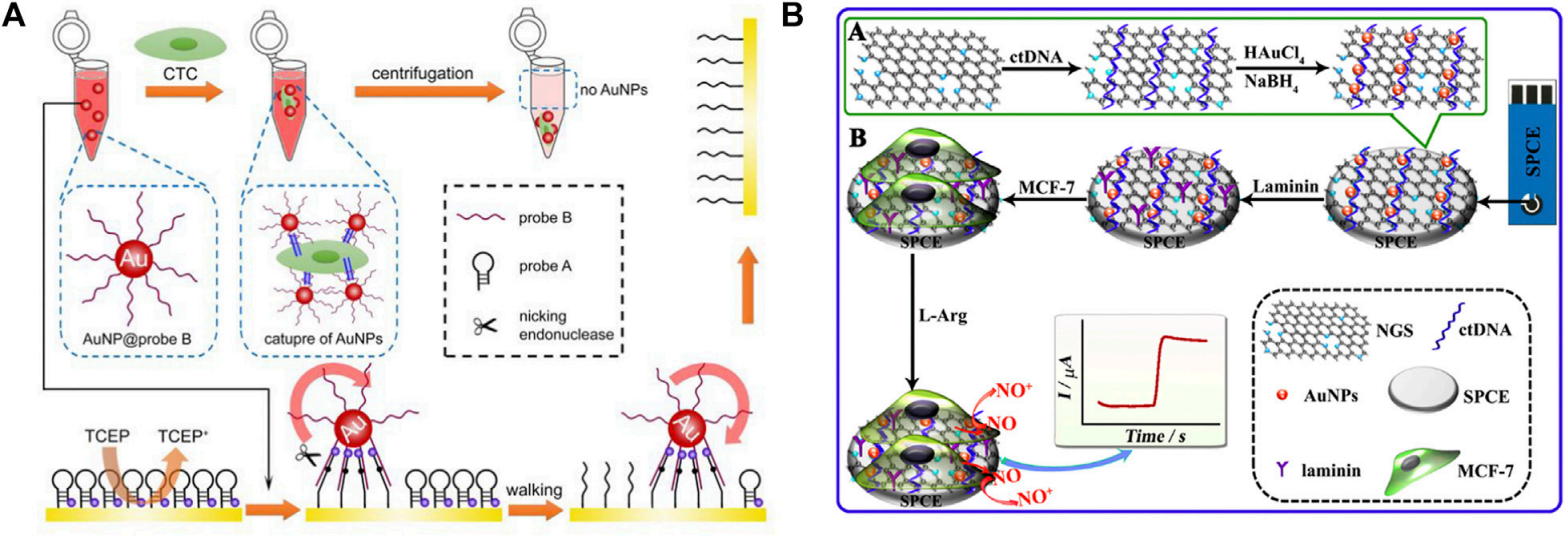
Schematic of electrochemical biosensors **(A)** based on DNA walker and **(B)** nitrogen-doped graphene sheets (NGS) for detecting cells.

Electrochemical biosensors are widely used for biomolecule detection because they are portable, simple, easy to use, and cost-effective. Because of their excellent conductivity, high surface areas, and catalytic properties, AuNPs as ideal candidates for electrochemical-based detection strategies offer new opportunities for highly sensitive biomarker detection. Miao and Tang. (2019) employed DNA-AuNPs for circulating tumor cell (CTC) detection, which provides a novel strategy for the sensitive and accurate quantification of CTC (Figure 11A). Owing to the large specific surface area of AuNPs, numerous walker strands are easily modified on AuNPs. The aptamer sequence can especially recognize the transmembrane receptor protein of CTC, and the AuNPs can be enriched on the surface of cells. The aptamer shows extremely excellent sensitivity and because multiple walker stands are modified on a single AuNP, hybridization with several tracks on the electrode occurs simultaneously for following nicking endonuclease-catalyzed cleaving, which provides excellent signal amplification. This strategy based on DNA-AuNPs can achieve the resolution of a single-cell level with excellent selectivity. The ability to distinguish CTC in blood samples also demonstrates its promising use in cancer diagnosis.

Dou et al. (2019) developed a novel platform for living cancer cells, which highly dispersed AuNPs on nitrogen-doped graphene sheets (NGS). The sensing platform could improve the electrocatalytic ability to detect nitric oxide (NO) released from live cancer cells (Figure 11B). Because of the synergistic enhancement of the catalytic activities of AuNPs and NGS as well as the high dispersion of AuNPs, such a nanocomposite shows significant electro-oxidation capability toward NO, leading to a highly sensitive with an extremely low detection limit for NO *in vitro*.

### 3.2 Bioimaging and therapy based on DNA-AuNPs

The imaging technique of cancer cells is of great significance for early cancer diagnosis and targeted therapy. DNA-AuNPs have attracted considerable attention because of their high loading capacity and excellent biocompatibility. Bioimaging and therapy for disease cells based on DNA-AuNPs is promising new single-entity techniques that possess significant advantages in early cancer diagnosis and treatment.

#### 3.2.1 Bioimaging

**FIGURE 12:**
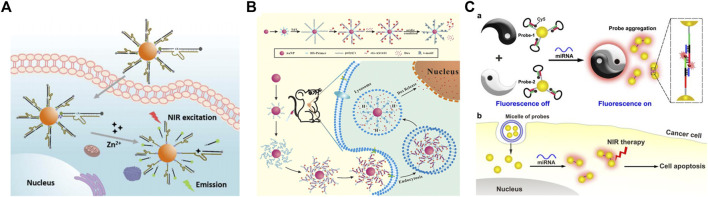
Schematic of the application of DNA-modified AuNPs in **(A)** bioimaging **(B)** drug delivery, and **(C)** photothermal therapy. Reprinted with permission from refs 95, 98, 99.

DNA-AuNPs are ideal candidates for bioimaging for *in situ* detection and monitoring of various molecules because AuNPs have excellent fluorescence effects and biocompatibility. Yang et al. (2018) developed an RNA-cleaving DNAzyme-based TP imaging probe for Zn^2+^ detection in living cells, which uses DNA-AuNPs for the imaging of intracellular metal ions. As shown in Figure 12A, they modified the Zn^2+^-specific DNAzyme with a fluorophore, which can be quenched by both AuNPs and the molecular quencher, and recognized intracellular Zn^2+^ in the surface of AuNPs. This strategy can be generally applied to detect other molecules in biological systems under TP imaging with highly sensitive and low phototoxicity.

Other works based on DNA-AuNPs were reported for miRNA *in situ* imaging in living cells. For example, a novel strategy for miRNA detection was developed by Ye et al. (2021). In this strategy, a near-infrared (NIR)-photoactivatable system, which was powered by endogenous adenosine triphosphate, was developed for *in situ* miRNA imaging with spatial and temporal resolutions. Chen et al. (2021) also developed a specific and sensitive platform for cancer-related miRNA in living cells, which is desirable for cancer diagnosis and treatment.

#### 3.2.2 Drug delivery

Because of the non-toxic and biocompatible characteristics of DNA-AuNPs, which have attracted increasing attention in the drug carriers, Sun et al. (Sun et al., 2019b) developed a novel carrier based on DNA-AuNPs for tumor-targeted delivery and site-specific release of the anticancer drug as an efficient method to overcome the side effects of traditional cancer therapy (Figure 12B). In this strategy, terminal deoxynucleotidyl transferase-catalyzed DNA extension reaction is used to prepare a thick DNA layer on the surface of AuNPs by extending long poly C) sequences from DNA primers immobilized on AuNPs. The poly C) extension products can then hybridize with G-rich oligonucleotides to give CG-rich DNA duplexes for loading the anticancer drug doxorubicin and multiple aptamers. The Dox-loaded DNA carrier can be efficiently internalized in cancer cells by recognizing multiple aptamers with the protein on the surface of cancer cells and achieving the burst release of drugs in acidic organelles due to the i-motif formation-induced DNA duplex destruction. This pH-responsive drug carrier was demonstrated to be promising for highly efficient delivery of Dox and selective killing of cancer cells in both *in vitro* and *in vivo* experiments, showing great potential in drug delivery.

#### 3.2.3 Photothermal therapy

Photothermal therapy is another application based on DNA-AuNPs for the therapy of cancer cells. Qian et al. (2016) developed a binary system for miRNA-21-targeted imaging and photothermal treatment in single cells. The binary system comprises a pair of thiol DNA probes, which are modified on AuNPs and encapsulated in the liposome for cell delivery (Figure 12C). The DNA-AuNPs were functionalized with Cy5-marked molecular beacon, which could be opened upon miRNA-21-triggered hybridization and turn on the fluorescence of Cy5 for localizing miRNA-21. The miRNA-induced aggregation shifts the absorption of AuNPs to NIR, which can be observed under dark-field microscopy and further used for the following photothermal therapy. Hence, MCF-7 breast cancer cells can be selectively killed *via* NIR irradiation. The proposed strategy provides a novel application of DNA-AuNPs for intracellular miRNA analysis and targeted treatment against cancer. In addition, Chen et al. (2020) proposed a furin‐instructed intracellular gold nanoparticle aggregation strategy and a furin‐responsive gold nanoparticle platform (AuNP@1) is designed for effective PTT of cancer both *in vitro* and *in vivo*. And Zhang et al. (2019b) developed a novel immunological AuNP through intracellular generation and exocytosis for combinatorial PTT and immunotherapy, and significantly improved overall survival of mice.

## 4 Conclusion and perspective

In this study, we have discussed various modification methods of DNA-AuNPs designed and successfully applied in the biomedical field. With the emergence of novel modification methods of DNA-AuNPs, more convenient conditions for applying them have emerged. These nanomaterials have paved the way for developing highly sensitive and efficient tools for the biomedical field. Although many applications based on DNA-AuNPs have been successfully established, they are still facing several challenges. First, the modification methods of DNA-AuNPs combined with new technologies are fairly insufficient. Second, the variation of AuNPs in size, shape, concentration, and dispersion buffer could cause significant variation in the modification effect, yielding poor reproducibility of the platforms based on DNA-AuNPs; hence, uniform modification methods of DNA-AuNPs are urgently needed. Third, most DNA-AuNP-based assays are just at the laboratory level and clinical applications have not been realized. They need to be combined with more clinical applications for disease diagnosis and therapy.
